# In memoriam: Dr. José Alberto Alvarenga (1948–2025)

**DOI:** 10.1055/s-0046-1817048

**Published:** 2026-03-11

**Authors:** Delson José da Silva

**Affiliations:** 1 Universidade Federal de Goiás, Hospital das Clínicas, Unidade do Sistema Neurológico, Neurologia e Neurocirurgia, Goiânia GO, Brazil.

**Figure 1:**
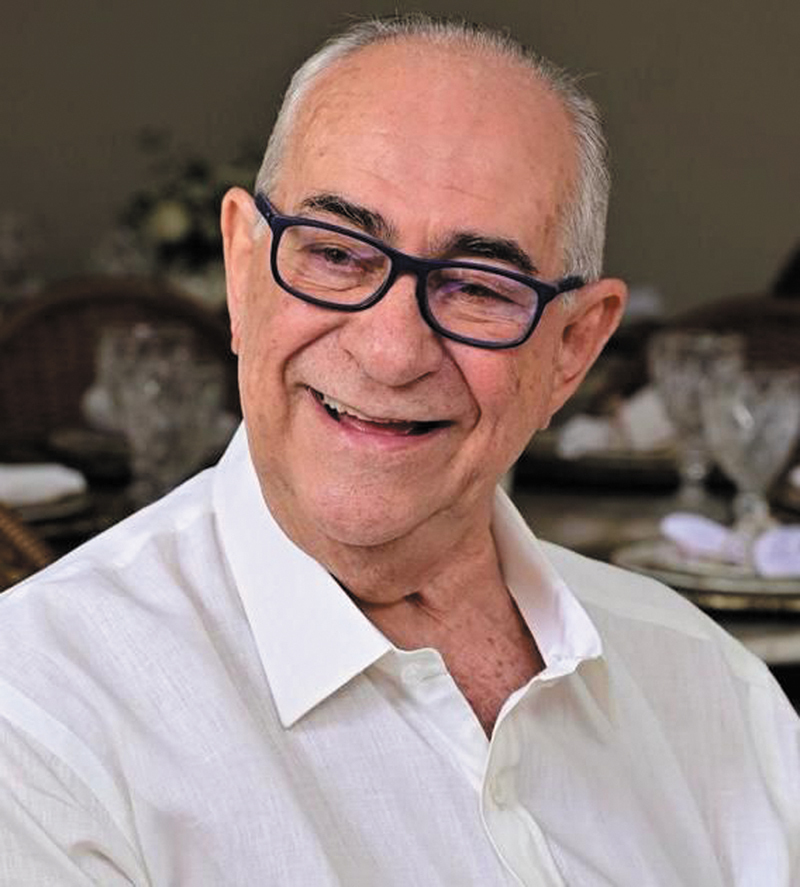
Dr. José Alberto Alvarenga.

Sometimes, no matter how much effort one may pour into writing a proper eulogy, the words on the page insist on feeling like falling short of the individual's legacy. This is one of these times.

On August 31, 2025, at the age of 76, the medical community of the state of Goiás–and indeed of Brazil–lost one of its most distinguished neurologists and neurophysiologists, Dr. José Alberto Alvarenga. His passing leaves an immeasurable void; yet, his legacy of knowledge, ethics, compassion, and devotion to medicine will live on in the hearts of those he touched and will continue to inspire generations to come. Widely known as Dr. Alvarenga, he built a solid and respected career marked by excellence, distinguished by his dedication to patient care and to the education of future physicians. His professional and academic trajectory reflects his unwavering commitment to continuous improvement and the pursuit of knowledge.

Dr. Alvarenga began his medical studies in 1967 in Goiânia, capital city of the state of Goiás, at Universidade Federal de Goiás (UFG), where he graduated in 1972. In the following years, he moved to São Paulo, the largest city in Brazil, where he undertook his neurology residency at Hospital dos Servidores Públicos and specialized in clinical neurophysiology under the Brazilian Society of Neurophysiology (Sociedade Brasileira de Neurofisiologia, in Portuguese). It did not take long for Dr. Alvarenga to come back to Goiânia, as he was already practicing neurology at Santa Casa de Misericórdia de Goiânia by 1976 and at Hospital Geral de Goiânia (HGG) by 1985.

He returned to UFG in 1989—now as a professor—and soon after joined the permanent faculty of the Pontifícia Universidade Católica de Goiás. Always the learner and driven by a relentless pursuit of knowledge, Dr. Alvarenga enrolled in the master's program at the UFG's Institute of Tropical Pathology and Public Health in 2006 and successfully defended his dissertation in 2008.

It is easy to assess Dr. Alvarenga's natural leadership by revisiting the numerous positions he held throughout his career. To mention just a few, he served as Head of Neurology at Hospital Estadual Dr. Alberto Rassi (formerly known as HGG - Hospital Geral de Goiânia), as Chief of the Clinical Neurology both at Santa Casa de Misericórdia and HGG, Director of the Regional Council of Medicine of Goiás for several consecutive terms, and as Dean of the Medical School at the Universidade de Rio Verde, whose Student's Union proudly bears his name.

His lifetime of dedication and service to Neurology earned him wide recognition among his peers, as Dr. Alvarenga was honored at the 3 _rd_ Congress of Neurology of Goiás, in 2007, and at the 29th Brazilian Congress of Neurology, in 2021. He was a Full Member of the Brazilian Academy of Neurology (Academia Brasileira de Neurologia, in Portuguese) and held Chair n° 39 of the Goiana Academy of Medicine (Academia Goiana de Medicina, in Portuguese). Even in his later years, his passions sang louder than any prospect of inaction, as he became a member of HGG administration after retiring from public service.

However important these achievements are, his greatest legacy was certainly the founding of the Neurology Residency Program in HGG in 1985, which was later merged with Santa Casa de Misericórdia's residency program. Over the following 4 decades, Dr. Alvarenga led the training of more than 100 neurologists, who now practice throughout Goiás, across Brazil, and beyond, each carrying forward his remarkable legacy.

Personally, I had the unique opportunity of being his first resident back in 1985, but that distinguished honor pales in the face of the privilege of having Alvarenga—as I fondly called him—as a close friend. Every occasion we shared our thoughts, Alvarenga always met any daring idea with generous and enthusiastic support—some of them even became shared goals and endeavors within the Brazilian Academy of Neurology.

Alvarenga's dedication, ethics, and love for life will continue to inspire all who had the privilege of knowing and learning from him. Yet the sorrow of losing such an extraordinary person is tempered by gratitude for the invaluable lessons he imparted. In that sense, Alvarenga is survived by his beloved wife, daughters, granddaughters, relatives, friends—and countless disciples.

